# Chemical Composition, Nutritional Profile, and Bioactive Properties of *Diplotaxis tenuifolia*, a Health-Promoting Food

**DOI:** 10.3390/molecules31030417

**Published:** 2026-01-26

**Authors:** Sandrine Ressurreição, Lígia Salgueiro, Artur Figueirinha

**Affiliations:** 1 University of Coimbra, Faculty of Pharmacy, 3000-548 Coimbra, Portugal; sandrine@esac.pt (S.R.); amfigueirinha@ff.uc.pt (A.F.); 2 Polytechnic University of Coimbra, Coimbra Agriculture School, 3045-601 Coimbra, Portugal; 3 Chemical Engineering and Renewable Resources for Sustainability (CERES), University of Coimbra, 3030-790 Coimbra, Portugal; 4 Associated Laboratory for Green Chemistry (LAQV) of the Network of Chemistry and Technology (REQUIMTE), University of Coimbra, 3000-548 Coimbra, Portugal

**Keywords:** food system, sustainability, nutraceutical value, bioactive compounds, phytochemicals, antioxidant potential

## Abstract

*Diplotaxis tenuifolia* (Brassicaceae), valued for its culinary use and bioactive potential, has not yet been comprehensively characterized in terms of its chemical composition and biological properties. This study investigated the nutritional profile, phytochemical composition, and antioxidant activity of *D. tenuifolia* cultivated in Portugal. The leaves contain substantial levels of essential minerals, particularly calcium, potassium, magnesium, iron, manganese, and chromium, while heavy metal levels were below regulatory safety limits. The nutritional profile also revealed high dietary fiber content, enriched glutamic and aspartic acids in the protein fraction, and α-linolenic acid as the predominant fatty acid. Phenolic compounds were most efficiently extracted by boiling them in 80% methanol, yielding the highest total phenolic (125.41 mg gallic acid equivalents g^−1^) and flavonoid contents (3.72 mg quercetin equivalents g^−1^). HPLC-PDA-ESI-MS^n^ analysis enabled the detailed characterization of phenolic acids, flavonol glycosides, and glucosinolates, highlighting the first report of sulfoglucobrassicin in *D. tenuifolia*. Additionally, 6-methylsulfonyl-3-oxohexyl-glucosinolate, proline, pipecolic acid, glucaric acid, eicosanoic acid, 9,10,12,13-tetrahydroxy-octadecanoic acid (sativic acid) and 9,12,13-trihydroxyoctadec-10-enoic acid were described for the first time in this species. The extract exhibited also antioxidant activity, with ABTS IC_50_ 57.54 ± 0.18 µg mL^−1^, DPPH IC_50_ 302.73 ± 2.36 µg mL^−1^, and FRAP 752.71 ± 4.59 µmol eq. Fe(II) g^−1^. These findings establish *D. tenuifolia* as a nutritionally rich plant and a promising source of natural antioxidants for nutraceutical and pharmaceutical applications.

## 1. Introduction

The Brassicaceae is a botanically highly diverse plant family comprising 354 genera, several of which are integral to human diets [1,2]. Although the genus *Diplotaxis* remains less prominent and far less studied than other Brassicaceae genera, it has recently gained scientific attention due to its species diversity. Within this taxa, *Diplotaxis tenuifolia* (L.) DC., known as perennial wall-rocket, is the only species subjected to extensive agricultural production and is valued both for culinary purposes and for its bioactive compounds of pharmaceutical interest. Native to Europe, North Africa, and the Near East, it is rich in bioactive compounds such as phenolic compounds and glucosinolates, and has been consumed as a vegetable since at least the 19th century. Particularly in Italy, it became a traditional ingredient in fresh salads, pizza, sauces, soups, and in the preparation of the digestive and diuretic liqueur *rucolino*, valued for its slightly peppery flavor, culinary versatility, and health-promoting properties [3]. The characteristic bitter or pungent taste of the leaves is attributed to glucosinolates, while their pronounced acrid aroma arises from volatile isothiocyanates, thereby linking their culinary appeal to the bioactive compounds responsible for their therapeutic potential [3,4]. Italy remains the main producer and consumer, with over 4000 hectares cultivated, although its popularity is increasingly expanding worldwide. Leaves are usually collected at an early vegetative stage, prior to flowering, within 20–100 days after planting or regrowth, allowing multiple harvests under favorable environmental conditions [3,4].

Concerning secondary metabolites, only flavonols and glucosinolates have been partially characterized in *D. tenuifolia* [4,5,6,7,8,9,10,11,12]. The available studies have preliminary examined several biological effects, suggesting relevant biological potential that warrants further investigation. Hypolipidemic activity was evaluated through pancreatic lipase inhibition using hydroethanolic extracts, with an IC_50_ value of 7.76 ± 0.08 mg/mL [13]. Antioxidant properties have likewise been investigated, although these studies are scarce and varied in their experimental approaches [10,14,15,16]. Cytotoxicity and antiproliferative effects were assessed in human colon carcinoma cells (Caco-2 cells) using the aqueous leaf extract in the MTT assay, suggesting that the extract may inhibit cell division and proliferation, an important feature in anticancer research [14]. Finally, anti-melanogenic properties were analyzed in B16F10 melanoma cells, showing inhibition of melanin production and tyrosinase activity [15]. However, most of these studies did not include a comprehensive phytochemical characterization of the extracts, relied solely on HPLC analyses, and did not employ complementary techniques such as LC–MS^n^, limiting compound identification and, consequently, the full understanding of the biological effects attributed to this species. Although several phytochemical studies have been reported, the nutritional composition and the broader biological activities of *D. tenuifolia* remain insufficiently explored, highlighting the need for comprehensive phytochemical analysis to identify bioactive compounds, ensure reproducibility, and establish meaningful correlations between chemical composition and biological effects. In this context, the present study aims to address these gaps by investigating *D. tenuifolia* samples cultivated and harvested in Portugal, assessing their nutritional profile directly and preparing multiple extracts for phytochemical characterization and evaluation of antioxidant activity. This integrated approach is intended to contribute to a clearer understanding of the species’ potential and to support future development of standardized and safe pharmaceutical or nutraceutical applications.

## 2. Results and Discussion

### 2.1. Food Safety and Nutritional Composition

Understanding the nutritional composition of foods is essential for assessing their dietary value and supporting a healthy diet. *D. tenuifolia*, the most widely cultivated and consumed species of the genus, has gained increasing attention due to its culinary use and growing consumption. However, data on its nutritional composition remain limited. To address this gap, the plant was subjected to a comprehensive nutritional analysis.

Ensuring food safety is of paramount importance. To promote safe cultivation practices, both the soil and irrigation water were thoroughly analyzed prior to planting to confirm compliance with established quality standards (Table A1 and Table A2). Plants of the Brassicaceae family are recognized for their ability to uptake and accumulate heavy metals [4,17]. However, this trait requires careful monitoring, as the accumulation of heavy metals in plant tissues may pose potential health risks.

For this purpose, the analysis began with the determination of the mineral, including both essential minerals and heavy metals, and the results are presented in Table 1.

Quantify potential contaminant levels, a thorough series of safety assessments was carried out on the harvested *D. tenuifolia* leaves, targeting key toxic elements, including cadmium, lead, and mercury. According to Commission Regulation (EU) No 2023/915 of 25 April 2023, the maximum allowable concentrations of these metals are 10 µg 100 g^−1^ for lead and 4 µg 100 g^−1^ for cadmium in brassicas and 10 µg 100 g^−1^ for mercury in food supplements and salt [18]. Analysis of the *D. tenuifolia* samples confirmed that all measured values were below these thresholds. Conducting such safety evaluations is critical for ensuring that the cultivated plants are free from health hazards and for safeguarding the nutritional quality of the final product. Maintaining strict monitoring of environmental conditions and contaminant levels is essential for the reliability of the experimental results and for the safe use of *D. tenuifolia* in food and dietary applications. To date, safety assessments remain unavailable for the vast majority of *Diplotaxis* species, highlighting the need for comprehensive investigations across the genus [4,19].

Concerning mineral composition, some mineral content data for *D. tenuifolia* have already been reported, showing concentrations of potassium 468 mg 100 g^−1^, calcium 309 mg 100 g^−1^, phosphorus 41 mg 100 g^−1^, and iron 5.20 mg 100 g^−1^ on a fresh weight basis. When expressed on a dry matter basis to allow direct comparison, the values obtained in this study differ from those previously described. Such variations are expected, as mineral content can be influenced by edaphoclimatic factors, cultivation practices, and the plant’s developmental stage.

*D. tenuifolia* contains substantial amounts of essential minerals, particularly calcium, potassium, magnesium, iron, manganese and chromium as established by Regulation (EU) No 1169/2011 of the European Parliament and Council dated 25 October 2011, which considers a mineral nutritionally significant if it provides more than 15% of the Nutrient Reference Value (NRV) [20].

Comparing the dry matter composition of *Eruca sativa*, the common *arugula* we usually consume, and *D. tenuifolia*, the wild arugula also marketed, some differences can be observed between the two species. *D. tenuifolia* shows a slightly higher ash content of 24.53 g 100 g^−1^, while *E. sativa* has 22.89 g 100 g^−1^. It also presents significantly higher calcium levels at 3952.03 mg 100 g^−1^, compared to 1927.71 mg 100 g^−1^ in *E. sativa*. Potassium and phosphorus contents are lower in *D. tenuifolia*, with 2420.09 mg 100 g^−1^ and 473.16 mg 100 g^−1^, respectively, whereas *E. sativa* contains 4457.83 mg 100 g^−1^ and 626.51 mg 100 g^−1^. Magnesium, iron, and zinc levels in *D. tenuifolia* are similar to those in *E. sativa* [21].

When compared to other edible species within the same genus, *D. tenuifolia* exhibits higher concentrations of calcium, potassium, phosphorus and iron compared to *D. erucoides* and higher potassium levels than *D. muralis* [19,22]. In comparison with *D. simplex*, *D. tenuifolia* contains higher levels of both calcium and magnesium [23].

Subsequently, the remaining nutritional parameters were assessed, with the results shown in Table 2.

Some data reported in other studies provide an overview of the nutritional composition of *D. tenuifolia*. Available reports indicate, the plant contains 91.0 g 100 g^−1^ moisture, 1.3 g 100 g^−1^ ash, 2.6 g 100 g^−1^ protein, 0.3 g 100 g^−1^ lipids, 0.9 g 100 g^−1^ crude fiber, and 4.8 g 100 g^−1^ total carbohydrates (including fiber), providing 28.7 kcal 100 g^−1^ in raw matter [4,24]. However, when expressed on a dry matter basis to enable comparison, the results obtained in this study differ from those reported in the literature. These differences are likely due to edaphoclimatic conditions, soil properties, and cultivation practices, which can influence the plant composition. Highlighting such variability is important because the relevance of the values depends on the intended use, whether for nutritional quality, bioactive compound content, or other applications. Therefore, the reported figures should be interpreted as general references, rather than absolute standards [25].

Comparing the dry matter composition of *Eruca sativa* and *D. tenuifolia*, carbohydrate levels are similar, measuring 26.5 g 100 g^−1^ in *E. sativa* and 25.7 g 100 g^−1^ in *D. tenuifolia*. However, *E. sativa* stands out for its significantly higher protein content at 31.3 g 100 g^−1^ compared to 12.66 g 100 g^−1^ in *D. tenuifolia*, and higher lipid content at 8.4 g 100 g^−1^ compared to 3.45 g 100 g^−1^. *D. tenuifolia*, on the other hand, has a much higher dietary fiber content, reaching 33.67 g 100 g^−1^ compared to 19.3 g 100 g^−1^ in *E. sativa* [21]. The intake of dietary fiber contributes significantly to digestive health. The majority of fiber is insoluble, supporting regular bowel movements by increasing stool volume and easing its passage through the intestines, which helps prevent constipation [26]. In contrast, soluble fiber, though present in smaller quantities, is readily metabolized by intestinal bacteria, enhancing microbial diversity and gut balance. This microbial fermentation produces short-chain fatty acids (SCFAs), compounds that help maintain gut health and may protect against digestive disorders [26].

The amino acid composition of the protein fraction in *D. tenuifolia* leaves in dry matter is characterized by high levels of glutamic and aspartic acids and moderate amounts of leucine, lysine, and valine. This composition indicates that *D. tenuifolia* may serve as a source of essential amino acids, potentially contributing to dietary protein quality and overall nutritional intake. This is the first study reporting the amino acid profile of *D. tenuifolia*.

Comparatively, in *E. sativa*, glutamic acid and aspartic acid are also the predominant amino acids [27]. Similar patterns are observed in other Brassicaceae species, where glutamic and aspartic acids represent the most abundant amino acids, as seen in kale (*Brassica oleracea* var. *acephala*) and broccoli (*Brassica oleracea* var. *italica*) [19,28,29]. Within the genus *Diplotaxis*, *D. tenuifolia* exhibits a higher glutamic acid concentration than *D. muralis*, the only other species in the genus with a fully characterized amino acid profile [19].

The fatty acid composition of the studied species is presented in Table 3. To date, no published data are available regarding the fatty acid composition of *D. tenuifolia*. In this study, *D. tenuifolia* showed α-linolenic acid (C18:3 n-3) as the predominant fatty acid, accounting for 38.22% of total fatty acids. Similarly, in other species of the *Diplotaxis* genus, α-linolenic acid is the major fatty acid in the leaves of *D. muralis* (40.58%) and *D. simplex* (25.40%) [19,23]. In contrast, the non-flowering aerial parts of *D. erucoides* exhibit linoleic acid (C18:2 n-6) as the main fatty acid. representing 29.10% of the total [30]. Both α-linolenic acid and linoleic acid are unsaturated and essential fatty acids, and their dietary intake promotes cardiovascular health and exerts anti-inflammatory effects [31].

### 2.2. Secondary Metabolites

Secondary metabolites, such as phenolic compounds, particularly flavonols and phenolic acids, as well as glucosinolates, are associated with a wide range of biological activities, including antioxidant, anti-inflammatory, antibacterial, hypoglycemic, hypolipidemic, cytotoxic and antiproliferative effects [4]. The extent of these bioactivities can be influenced by several factors, notably the extraction conditions, which are crucial for defining the chemical profile of the extracts. To capture a diverse profile of these compounds, leaves of *D. tenuifolia* were processed using different extraction techniques: boiled in with 80% methanol, maceration in 100% ethanol and 50% ethanol, maceration in water and aqueous infusion. The extraction process resulted in yields of 1.0%, 1.3%, 2.1%, 4.5% and 5.5% for the respective methods. Quantification of total phenolic and flavonoid contents was carried out, and the outcomes are summarized in Table 4.

The choice of extraction method strongly affected the total phenolic and flavonoid contents in *D. tenuifolia* extracts. Among all approaches, boiled in with 80% methanol was the most effective, yielding 125.41 mg of gallic acid equivalents (GAE) per gram for phenolic compounds and 3.72 mg of quercetin equivalents (QE) per gram for flavonoids. Although the phenolic and flavonoid composition of this species had not been studied before, the results are consistent with other *Diplotaxis* species. Methanolic leaf extracts of *D. muralis* contained 68.36 mg GAE g^−1^ phenolics and 3.50 mg QE g^−1^ flavonoids, while those of *D. simplex* and *D. harra* displayed even lower total phenolics, 4.79 and 5.47 mg GAE g^−1^, respectively [19,32]. These results indicate that *D. tenuifolia*, particularly when extracted in 80% methanol, provides superior levels of phenolic and flavonoid compounds, emphasizing the influence of both species and extraction method.

Based on the high content of total phenolic compounds and flavonoids, HPLC-PDA-ESI-MS^n^ analysis was carried out on the *D. tenuifolia* leaf extract obtained by boiling it in 80% methanol. This approach allowed for the precise identification of individual compounds, which are presented in Table 5.

#### 2.2.1. Phenolic Acids and Flavonols

Compound **3** shows the [M + H]^+^ ion at *m*/*z* 123, with the base peak corresponding to the intact molecular ion. A minor fragment at *m*/*z* 105 arises from the loss of a water molecule (−18 Da) or a typical rearrangement of the aromatic ring. This fragmentation pattern was previously related to benzoic acid [34].

Compound **7** shows the [M + H]^+^ ion at *m*/*z* 167, with the base peak at *m*/*z* 126, resulting from the loss of a neutral fragment from the carboxylic acid group. Additional fragments at *m*/*z* 149 and 85 reflect typical cleavages of the side chain in dihydro-coumaric acid. The observed fragmentation pattern confirms the identification of this compound as dihydro-coumaric acid (phloretic acid), previously identified by [32,33].

Compound **10** exhibited UV absorption bands with a maximum near 252 nm and a weaker band around 332 nm, a pattern consistent with a 3-*O*-substituted flavonol monohydroxylated on ring B, such as kaempferol. Compound **10** displayed a deprotonated molecular ion at *m*/*z* 609, with major fragments at *m*/*z* 447 and 285, reflecting sequential losses of sugar moieties and formation of the kaempferol aglycone. In positive ion mode, the [M + H]^+^ ion at *m*/*z* 611, with fragments at *m*/*z* 449 and 287 from glycosidic bond cleavage and the kaempferol aglycone, confirms Compound **10** as kaempferol-3-*O*-dihexoside [19,42].

Compound **11** exhibits a typical UV spectrum of a 3-*O*-substituted flavonol, similar to those of isorhamnetin and rhamnetin. It shows the [M + H]^+^ ion at *m*/*z* 641, with major fragments at *m*/*z* 479 and 317. The base peak at *m*/*z* 317 corresponds to the rhamnetin aglycone after the loss of sugar moieties, while the minor fragment at *m*/*z* 302 results from the loss of a methyl group (−15 Da). The mass spectrum shows a major peak at *m*/*z* 317 and a smaller one at *m*/*z* 302, while isorhamnetin primarily loses a methyl group, giving a single prominent peak at *m*/*z* 302, supporting its identification as rhamnetin-3-*O*-dihexose [42,43].

Compounds **9**, **12**, and **13** showed UV spectra with maxima around 270 and 350 nm, indicating that they are quercetin derivatives glycosylated at the 3-position of the flavonoid backbone. Compound **9** shows, in negative ion mode, the [M − H]^−^ ion at *m*/*z* 833, with major fragments at *m*/*z* 787 (base peak), 574, and 760, reflecting losses of sugar moieties typical of dihexosylated flavonoids. In positive ion mode, the [M + H]^+^ ion is observed at *m*/*z* 789, with prominent fragments at *m*/*z* 465 (base peak), 627, and 303, corresponding to sequential cleavages of glycosidic bonds and the quercetin aglycone. The fragmentation pattern confirms the identification of this compound as quercetin-3-*O*-hexoside-dihexoside, as previously reported in [19]. Compound **13** displayed a similar fragmentation pattern, but with an additional loss of a deoxyhexose unit, allowing its identification as quercetin-3-*O*-deoxyhexose-hexose [19,42]. In contrast, Compound **12** presented a deprotonated molecular ion at *m*/*z* 993 ([M − H]^−^), which underwent sequential loss of hexosyl units to generate fragment ions at *m*/*z* 831 and *m*/*z* 669, and further fragmentation involving the elimination of a sinapoyl group followed by another hexose ultimately produced a fragment at *m*/*z* 301, supporting its tentative identification as quercetin-3,4′-diglucoside-3′-(6-sinapoyl-glucoside), as previously reported in [6,19].

Compound **14** shows the [M + H]^+^ ion at *m*/*z* 1019, with major fragments at *m*/*z* 1002 (base peak), 958, and 913, reflecting sequential losses of sugar and methoxycaffeoyl moieties typical of complex flavonoid glycosides. The fragmentation pattern is consistent with cleavages of the glycosidic bonds and the acylated sugar units. Compound **14** corresponds to quercetin-3,4′-diglucoside-3′-(6-methoxycaffeoyl-glucoside), as described by [8].

Compound **19** displays the [M − H]^−^ ion at *m*/*z* 1199, with the base peak at *m*/*z* 1137, corresponding to the loss of one sinapoyl-glucoside moiety. The fragmentation pattern is consistent with sequential cleavages of glycosidic and acylated sugar units. Based on these data, Compound **19** is identified as quercetin-3-(2-sinapoyl-glucoside)-3′-(6-sinapoyl-glucoside)-4′-glucoside, as previously reported in [38,42].

Compound **20** exhibits the [M − H]^−^ ion at *m*/*z* 1169, with the base peak at *m*/*z* 1007, indicating the loss of one feruloyl-glucoside moiety. Its fragmentation pattern also reflects sequential cleavages of glycosidic and acylated sugar units. Accordingly, Compound **20** is identified as quercetin-3-(2-feruloyl-glucoside)-3′-(6-sinapoyl-glucoside)-4′-glucoside, also previously reported in [38,42].

Previous studies on *D. tenuifolia* secondary metabolites have identified various flavonols in the leaf extracts [4,5,6,7,8,9]. Mono-, di-, and triglycosides of quercetin, isorhamnetin, and kaempferol were identified, with quercetin-3,3′,4-triglucoside and quercetin-3,4′-diglucoside-3′-(6-sinapoylglucoside) being the most frequently reported flavonols [4,7,47]. Compounds such as quercetin-3,4′-di-glucoside-3′-(6-methoxycaffeoyl-glucoside), quercetin-3-(2-sinapoyl-glucoside)-3′-(6-sinapoyl-glucoside)-4′-glucoside, and quercetin-3-(2-feruloyl-glucoside)-3′-(6-sinapoyl-glucoside)-4′-glucoside have also been repeatedly reported in *D. tenuifolia* [38,42]. In contrast, quercetin-3-*O*-hexoside-dihexoside, kaempferol-3-*O*-dihexoside, rhamnetin-3-*O*-dihexose, and quercetin-3-*O*-deoxyhexose-hexose are reported here for the first time. Benzoic acid and dihydro-coumaric acid (phloretic acid), two phenolic acids, are also reported here for the first time in *D. tenuifolia*.

#### 2.2.2. Glucosinolates

**Figure 1:**
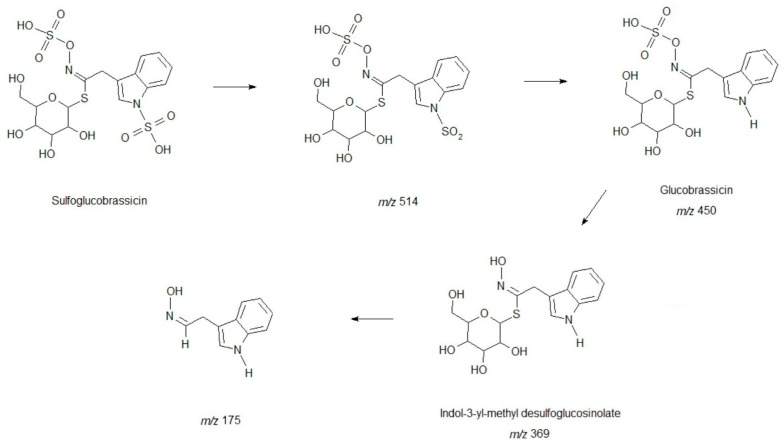
Proposed fragmentation pathway of sulfoglucobrassicin obtained in positive ion mode.

Compound **2** corresponds to the fragmentation of a glucobrassicin derivative containing an additional sulfate group. In positive ion mode ([M + H]^+^), the protonated molecular ion is observed at *m*/*z* 513. The base peak at *m*/*z* 175 corresponds to the indole nucleus, resulting from the complete loss of the glycosidic portion and both sulfate groups, highlighting the high stability of the aromatic indole ring. The fragment at *m*/*z* 369 corresponds to indol-3-yl-methyl desulfoglucosinolate, a key intermediate in the biosynthesis of glucosinolates. Other significant fragments, including *m*/*z* 450, 352, and 337, reflect sequential losses of the sugar and sulfate groups, as well as typical rearrangements of indol-3-yl glycoside derivatives, providing insight into the position and stability of the indole core and the glycosidic and sulfonated linkages. In negative ion mode ([M − H]^−^), the deprotonated molecular ion is observed at *m*/*z* 510. The most intense fragment at *m*/*z* 430 corresponds to the loss of one SO_3_ group (80 Da). The fragment at *m*/*z* 422 reflects the loss of HSO_4_, while the fragment at *m*/*z* 372 results from the combined loss of both sulfate groups and part of the glucose attached to the indole nucleus. Smaller fragments at *m*/*z* 268, 250, and 234 correspond to more extensive cleavages of the molecule, including elimination of the glycosidic portion and parts of the indole side chain. The observed fragmentation pattern, with sequential losses of both sulfate groups, glucose, and the indole nucleus, confirms that Compound **2** corresponds to sulfoglucobrassicin. The fragmentation of sulfoglucobrassicin is illustrated in Figure 1, showing the proposed fragmentation pathway.

Compound **4** corresponding to [M − H]^−^ of glucoraphanin is observed at *m*/*z* 436. The base peak at *m*/*z* 372 results from the loss of glucose moiety, which is typical for glucosinolate derivatives. Additional fragments include *m*/*z* 259 and 275, reflecting cleavages within the side chain and partial loss of the sulfinyl group. A smaller fragment at *m*/*z* 194 corresponds to the indole or aliphatic fragment remaining after sequential losses, consistent with typical fragmentation pathways of glucoraphanin reported in the literature [19,35,36].

Compound **8** corresponding to [M − H]^−^ of 6-methylsulfonyl-3-oxohexyl-glucosinolate is observed at *m*/*z* 494. The base peak at *m*/*z* 414 results from the loss of the glucose moiety, a common fragmentation pathway for glucosinolate derivatives. The fragment at *m*/*z* 252 corresponds to the cleavage of the side chain, including the sulfonyl and keto functionalities, reflecting characteristic fragmentation of aliphatic glucosinolates. The observed fragmentation pattern is consistent with the structure of 6-methylsulfonyl-3-oxohexyl-glucosinolate [11,34].

Several glucosinolates have been identified in *D. tenuifolia*, with glucosativin, glucoraphanin, and glucoerucin being the most frequently reported. Glucoraphanin had also been previously reported in the species, whereas sulfoglucobrassicin and 6-methylsulfonyl-3-oxohexyl-glucosinolate are reported here for the first time [4,6,10,11,12]. The pungent and spicy properties are largely attributed to isothiocyanates, formed when glucosinolates are enzymatically hydrolyzed by myrosinase [48]. Glucoraphanin undergoes enzymatic conversion to sulforaphane, an isothiocyanate responsible for the pungent and slightly bitter flavor characteristic of vegetables like broccoli and kale [6,48]. Similarly, 6-methylsulfonyl-3-oxohexyl-glucosinolate produces an isothiocyanate that gives a mild sulfurous and sharp taste, typical of cruciferous vegetables and reminiscent of mustard [49]. In contrast, glucobrassicin and sulfoglucobrassicin produce indole-derived compounds upon hydrolysis, such as indole-3-carbinol, which contribute milder, slightly bitter, and herbaceous flavors, adding subtle complexity to the overall taste profile of the species [50].

#### 2.2.3. Fatty Acids and Lipids

Compounds **15** and **16** show similar fragmentation patterns, characteristic of polyhydroxylated fatty acids. Compound **15** displays the [M − H]^−^ ion at *m*/*z* 347, with the base peak at *m*/*z* 329, resulting from the loss of a water molecule (−18 Da). Additional fragments at *m*/*z* 311, 293, 201, 171, and 129 reflect sequential water losses and cleavages along the carbon chain, consistent with the positions of the hydroxyl groups. Compound **16** shows the [M − H]^−^ ion at *m*/*z* 329, with the base peak at *m*/*z* 311, corresponding to the loss of water (−18 Da) from the hydroxyl groups, and additional fragments at *m*/*z* 293, 211, 171, and 275, representing sequential water losses and cleavages around the hydroxy and double-bond positions along the fatty acid chain. The observed fragmentation patterns confirm the identification of Compounds **15** and **16** as oxylipins, specifically 9,10,12,13-tetrahydroxy-octadecanoic acid (sativic acid) and 9,12,13-trihydroxyoctadec-10-enoic acid, respectively [42,44].

Compound **17** shows the [M − H]^−^ ion at *m*/*z* 311, with the base peak corresponding to the intact molecular ion. A minor fragment at *m*/*z* 293 arises from the loss of water (−18 Da), which is typical for long-chain saturated fatty acids. Compound **17** has previously been identified as eicosanoic acid (arachidic acid), as reported in [19,45].

This is the first report of these compounds in *D. tenuifolia*. Within the *Diplotaxis* genus, arachidic acid has previously been identified only in the flowers of *D. simplex* and the leaves of *D. muralis* while 9,12,13-trihydroxyoctadec-10-enoic acid has been reported in the aerial parts of *D. erucoides*. Other oxylipins have been identified in the aerial parts of *D. erucoides* and the leaves of *D. muralis* [4,19,23,42]. Arachidic acid (eicosanoic acid), contributes to membrane architecture and is involved in lipid metabolic pathways that influence stress signaling in plants [51]. Hydroxylated oxylipins are products of oxygenated fatty acid metabolism and act as plant signaling molecules, modulating defense responses and adaptive processes under environmental stress [52,53]. These lipid-derived metabolites thus play important roles in physiological regulation and metabolic adjustments in response to biotic and abiotic cues [51,52,53].

#### 2.2.4. Amino Acids

Compounds **5** and **6** display [M + H]^+^ ions and fragmentation patterns typical of amino acids. Compound **5** shows the [M + H]^+^ ion at *m*/*z* 116, with the base peak corresponding to the molecular ion. The fragmentation is simple, as expected for a small amino acid, and the spectrum is dominated by the intact protonated molecule. Compound **5** is therefore identified as proline [37].

Compound **6** shows the [M + H]^+^ ion at *m*/*z* 130, with a minor fragment at *m*/*z* 84, corresponding to the loss of part of the piperidine ring. The fragmentation pattern is consistent with the structure of a cyclic amino acid. Compound **6** is therefore identified as pipecolic acid, previously identified by [38]. This is the first report of these compounds in *D. tenuifolia*.

#### 2.2.5. Sugar Acids

Compound **1** at *m*/*z* 209 corresponds to the deprotonated molecular ion [M − H]^−^ and represents the base peak, indicating a stable anion. Fragment ions at *m*/*z* 191 (loss of H_2_O) and *m*/*z* 165 (loss of CO_2_) reflect typical cleavages of polyhydroxy dicarboxylic acids. Therefore, the observed fragmentation pattern supports the identification of the molecule as glucaric acid, previously described by [33]. This is the first report of this compound in *D. tenuifolia*.

### 2.3. Antioxidant Activity

*D. tenuifolia* is rich in characteristic bioactive compounds such as quercetin, kaempferol, and isorhamnetin *O*-glycosides, together with glucosinolates, all of which are known for their diverse biological properties. Given the presence of these compounds, we investigated the antioxidant capacity of *D. tenuifolia* to evaluate its potential health-promoting effects. The antioxidant properties of the extracts were examined using ABTS, DPPH, and FRAP assays, and the results are summarized in Table 6.

The extract of *D. tenuifolia* boiled in methanol (80%) exhibited the highest antioxidant activity, with IC50 values of 57.54 µg mL^−1^ for ABTS and 302.73 µg mL^−1^ for DPPH, and a FRAP value of 752.71 µmol Fe(II) equivalents per gram. These results align with its rich phenolic and flavonoid content. The ABTS assay, responsive to both polar and nonpolar compounds, showed the lowest IC50, underscoring the dominant role of phenolics in antioxidant activity. In contrast, the DPPH assay, more sensitive to nonpolar molecules, yielded a higher IC50, indicating relatively lower efficiency. FRAP measurements further confirmed the presence of strong reducing agents, likely phenolic compounds, that contribute to free radical neutralization [19].

Antioxidant activity of 80% methanol extracts from *D. tenuifolia* leaves, obtained by solid–liquid extraction assisted by homogenization (Ultra-Turrax), from plants grown under different greenhouse films and exposed post-harvest to UV-B for 45–660 s, ranged from 1276 to 2573 mg TE/100 g D.W. in the ABTS assay and from 988 to 1821 mg TE/100 g D.W. in the DPPH assay [16]. When compared and when converted to the same units, the extract boiled in methanol (80%) presented higher values for the ABTS assay and values within the reported range for the DPPH assay. These results may be explained by differences in extraction method and plant production conditions. Currently, there are no studies on the antioxidant activity, with investigations limited to aqueous extracts, in which the FRAP assay showed values ranging from 4.13 to 11.02 mmol kg^−1^ fresh weight (fw) [3,14].

Other species of the *Diplotaxis* genus, such as *D. harra*, *D. simplex*, *D. erucoides* and *D. muralis*, have also been evaluated [19,42,54,55]. In ABTS assays, IC50 values ranged from 78.81 to 929 µg mL^−1^, while in DPPH assays, results ranged from 135.13 to 5470 µg mL^−1^. For the FRAP assay, values were between 24.42 and 731.20 µmol eq. Fe(II) g^−1^. In ABTS and FRAP assays, the extract boiled in methanol (80%) of *D. tenuifolia* showed the best results, reflected in lower IC50 values. In the DPPH assay, the observed values were consistent with previously reported data. Under similar extraction conditions, *D. tenuifolia* extracts demonstrated superior performance compared to *D. muralis*, except for ethanol maceration at 50% in the ABTS assay (112.14 µg mL^−1^), and ethanol maceration at 100%, which exhibited a lower IC50 in the DPPH assay (547.72 µg mL^−1^) and a higher value in the FRAP assay (410.05 µmol eq. Fe(II) g^−1^). Overall, these findings emphasize the antioxidant potential of *D. tenuifolia* extracts, supporting their potential use as valuable natural antioxidants for food, nutraceutical, or pharmaceutical applications.

## 3. Material and Methods

### 3.1. Plant Material

For this study, certified seeds of *Diplotaxis tenuifolia* (L.) DC. were obtained from the Saflax brand (Lübeck, Germany), registered in Germany under the code DE-NW-4103359 and internally identified by the supplier as 18732. The seeds were initially germinated in small compartments filled with peat. Once seedlings emerged, they were carefully transplanted into a final plot of approximately 30 m^2^, which had been previously evaluated to ensure that both soil and water quality met the necessary conditions for healthy crop development (Table A1 and Table A2). Soil was analyzed following the methodologies described in [56,57], and water quality was assessed using test kits in combination with a DR3900 spectrophotometer (Hach, Loveland, CO, USA). Leaf harvesting was conducted in June 2024, prior to the onset of flowering, to ensure that leaves were collected while still young and tender. A plant specimen was preserved and deposited in the herbarium of the Faculty of Pharmacy, University of Coimbra for scientific documentation.

After the harvest, leaf coloration was assessed using a Chroma Meter CR-400 (Konica Minolta, Tokyo, Japan), measuring both the upper and lower surfaces of the leaves. The results, recorded in the CIE Lab color space, showed values of L* = 34.2, a* = −11.0, and b* = 9.2, indicating a predominantly dark tone with green and yellow tones. Following harvest, a portion of the leaf material was allocated for nutritional analysis, while the rest was freeze-dried for later phytochemical and antioxidant assays.

### 3.2. Food Safety and Nutritional Composition

The food safety and nutritional composition of *D. tenuifolia* leaves were evaluated following the protocols established by AOAC International [58]. Moisture content was determined using AOAC method 930.04, while ash content was measured according to AOAC method 930.05. Calcium, potassium, magnesium, phosphorus, sodium, iron, zinc, manganese, copper, chromium, and nickel were evaluated following AOAC method 975.03 by flame atomic absorption spectrometry (FAAS) (PerkinElmer PinAAcle 900 T, Waltham, MA, USA), and boron was determined by segmented flow analysis (CFA) (Skalar SAN++ System, Skalar Analytical B.V., Breda, The Netherlands). Phosphorus content was measured by spectrophotometry (Hitachi U-2000 spectrophotometer, Hitachi, Tokyo, Japan) according to AOAC method 948.09. Lead and cadmium concentrations were determined by graphite furnace atomic absorption spectrometry (GFAAS) (PerkinElmer PinAAcle 900 T, Waltham, MA, USA) following AOAC method 999.11 [59]. Total mercury in the samples was determined by direct thermal combustion (LECO AMA254 Mercury Analyzer, LECO Corporation, St. Joseph, MI, USA). Total lipids were determined using AOAC method 930.09. Crude protein was quantified using AOAC method 978.04 (conversion factor = 6.25). Crude fiber, as well as total and insoluble dietary fibers, were assessed using AOAC methods 930.10, 985.29, and 991.42, respectively (Total Dietary Fiber Assay Kit, Megazyme, Wicklow, Ireland). The contents of cellulose, hemicellulose, and lignin were calculated from measurements of neutral detergent fiber, acid detergent fiber, and acid detergent lignin using the Van Soest method [60]. Total carbon and sulfur were determined by high-temperature oxidation with infrared detection (Leco SC-144 DR Dual Range Sulfur and Carbon Analyzer, Leco, St. Joseph, MI, USA). Nitrogen-free extract, available carbohydrates and total carbohydrates and were calculated based on Food and Agriculture Organization (FAO) guidelines [61,62]. Energy content was expressed in both kcal and kJ per 100 g and calculated in accordance with EU Regulation No. 1169/2011 of the European Parliament and Council (25 October 2011) [20].

To determine the amino acid profile, the samples were first subjected to acid hydrolysis, and the resulting hydrolysates were then analyzed using UPLC-PDA (Waters^®^ Acquity UPLC, Waters Corporation, Milford, MA, USA), following the procedure outlined in [63]. The fatty acid composition was determined by converting the lipids into methyl esters (FAMEs) and analyzed by GC-FID using a Chrompack CP 9001 gas chromatograph (Middelburg, Zeeland, The Netherlands), as described in [64].

### 3.3. Extracts Preparation

Bioactive compounds were extracted from lyophilized plant material using boiling, maceration, and infusion. For 5 g of lyophilized material was refluxed in 80% methanol for 6 h. Maceration used 100% ethanol, 50% ethanol, or water, mixing 5 g of lyophilized material with 100 mL solvent and continuously agitating for 6 h. The infusion was prepared by adding 100 mL of boiling water to 5 g of lyophilized material and allowing it to steep for 15 min. Extracts were filtered, concentrated via rotary evaporation (Rotavapor R-114, Büchi, Flawil, Switzerland), lyophilized (FTS Systems, Stone Ridge, NY, USA), and stored at −22 °C, in the dark until analysis.

### 3.4. Identification of Secondary Metabolites by HPLC-PDA-ESI-MS^n^

Total phenolic and flavonoid contents were identified according to previously described methods [19,65,66]. HPLC-PDA-ESI-MS^n^ analysis was performed to characterize the phytochemical composition of extract of *D. tenuifolia* boiled in methanol (80%). Analyses were conducted using high-performance liquid chromatography with photodiode array detection and linear ion trap mass spectrometry (Thermo Fisher Scientific, Waltham, MA, USA), operating in both negative and positive ion modes, as described in [19]. The capillary temperature was 275 °C, with a capillary voltage of −35.00 V in negative mode and 40 V in positive mode. The source voltage was 5.00 kV.

### 3.5. Antioxidant Activity

Total antioxidant activity was evaluated using ABTS Assay (ABTS), 2.2-Diphenyl-1-Picrylhydrazyl Radical Assay (DPPH), and Ferric Reducing Antioxidant Power Assay (FRAP), as described in [65,67,68,69].

## 4. Conclusions

*D. tenuifolia* exhibits a noteworthy nutritional profile, being rich in essential minerals, dietary fiber, and amino acids. α-Linolenic acid was the predominant fatty acid in the lipid profile. Heavy metal concentrations were below safety limits, indicating that this sample is safe for human consumption. Among the extracts tested, the extract boiled in methanol (80%) showed the highest concentrations of phenolic compounds, along with the most pronounced antioxidant activity. HPLC-PDA-ESI-MS^n^ analysis allowed for the identification of a diverse array of flavonol glycosides and glucosinolates, highlighting the first report of sulfoglucobrassicin in this species. Additionally, 6-methylsulfonyl-3-oxohexyl-glucosinolate, proline, pipecolic acid, glucaric acid, eicosanoic acid, 9,10,12,13-tetrahydroxy-octadecanoic acid (sativic acid) and 9,12,13-trihydroxyoctadec-10-enoic acid were described for the first time in *D. tenuifolia*. These bioactive compounds likely contribute to the observed antioxidant activity and may play a role in the plant’s health-promoting properties. Taken together, these findings highlight the potential of *D. tenuifolia* as a source of bioactive compounds that could be further explored for functional foods. Further comprehensive studies on the bioavailability, metabolism, and detailed fractionation of its constituents are needed to better understand their biological activities and to support potential nutraceutical or pharmaceutical applications.

## Figures and Tables

**Table 1:** Essential mineral and heavy metal content of *D. tenuifolia* leaves.

Composition	Raw Matter	Dry Matter
Ash (g 100 g^−1^)	3.79 ± 0.01	24.53 ± 0.06
Calcium (mg 100 g^−1^)	610.09 ± 4.68	3952.03 ± 30.34
Potassium (mg 100 g^−1^)	373.60 ± 7.15	2420.09 ± 46.29
Magnesium (mg 100 g^−1^)	77.95 ± 2.14	504.95 ± 13.87
Phosphorus (mg 100 g^−1^)	73.04 ± 0.28	473.16 ± 1.84
Sodium (mg 100 g^−1^)	22.64 ± 0.76	146.67 ± 4.93
Iron (mg 100 g^−1^)	3.01 ± 0.04	19.49 ± 0.25
Zinc (mg 100 g^−1^)	0.61 ± 0.03	3.95 ± 0.21
Manganese (mg 100 g^−1^)	0.36 ± 0.01	2.34 ± 0.08
Boron (mg 100 g^−1^)	0.33 ± 0.01	2.16 ± 0.06
Copper (mg 100 g^−1^)	0.13 ± 0.01	0.82 ± 0.08
Chromium (µg 100 g^−1^)	96.08 ± 2.45	622.37 ± 15.85
Nickel (µg 100 g^−1^)	41.63 ± 2.35	269.67 ± 15.21
Lead (µg 100 g^−1^)	8.54 ± 0.05	55.34 ± 0.32
Cadmium (µg 100 g^−1^)	3.67 ± 0.02	23.76 ± 0.11
Mercury (µg 100 g^−1^)	0.15 ± 0.01	0.96 ± 0.03

**Table 2:** Proximate composition of *D. tenuifolia* leaves.

Composition	Raw Matter	Dry Matter
Moisture (g 100 g^−1^)	84.56 ± 0.06	-
Lipids (g 100 g^−1^)	0.53 ± 0.01	3.45 ± 0.06
Crude fiber (g 100 g^−1^)	1.15 ± 0.02	7.47 ± 0.10
Total dietary fiber (g 100 g^−1^)	5.20 ± 0.01	33.67 ± 0.07
Insoluble dietary fiber (g 100 g^−1^)	4.52 ± 0.03	29.25 ± 0.17
Soluble dietary fiber (g 100 g^−1^)	0.68 ± 0.01	4.42 ± 0.10
Neutral detergent fiber (g 100 g^−1^)	5.24 ± 0.02	33.96 ± 0.14
Acid detergent fiber (g 100 g^−1^)	1.82 ± 0.02	11.76 ± 0.12
Acid detergent lignin (g 100 g^−1^)	0.53 ± 0.02	3.46 ± 0.12
Cellulose (g 100 g^−1^)	0.63 ± 0.01	4.09 ± 0.07
Hemicellulose (g 100 g^−1^)	3.43 ± 0.02	22.19 ± 0.12
Lignin (g 100 g^−1^)	0.53 ± 0.02	3.46 ± 0.12
Total sulfur (g 100 g^−1^)	0.14 ± 0.00	0.89 ± 0.01
Total carbon (g 100 g^−1^)	4.60 ± 0.04	29.77 ± 0.25
Total nitrogen (g 100 g^−1^)	0.31 ± 0.00	2.03 ± 0.01
Protein (g 100 g^−1^)	1.95 ± 0.01	12.66 ± 0.05
Glutamic acid (mg 100 g^−1^)	340.91 ± 17.27	2207.95 ± 111.82
Aspartic acid (mg 100 g^−1^)	195.10 ± 11.31	1263.61 ± 73.25
Leucine (mg 100 g^−1^)	143.55 ± 4.54	929.73 ± 29.43
Lysine (mg 100 g^−1^)	109.80 ± 1.48	711.14 ± 9.58
Valine (mg 100 g^−1^)	103.59 ± 1.62	670.90 ± 10.48
Phenylalanine (mg 100 g^−1^)	101.36 ± 1.80	656.46 ± 11.68
Alanine (mg 100 g^−1^)	100.03 ± 2.18	647.84 ± 14.14
Serine (mg 100 g^−1^)	97.09 ± 7.93	628.79 ± 51.33
Proline (mg 100 g^−1^)	96.50 ± 5.93	624.98 ± 38.43
Glycine (mg 100 g^−1^)	92.02 ± 2.31	595.99 ± 14.99
Arginine (mg 100 g^−1^)	86.49 ± 3.97	560.17 ± 25.71
Threonine (mg 100 g^−1^)	78.02 ± 3.74	505.30 ± 24.22
Isoleucine (mg 100 g^−1^)	68.58 ± 0.32	444.18 ± 2.10
Tyrosine (mg 100 g^−1^)	67.98 ± 1.58	440.25 ± 10.26
Histidine (mg 100 g^−1^)	21.56 ± 2.94	139.67 ± 19.07
Methionine (mg 100 g^−1^)	33.88 ± 0.83	219.45 ± 5.36
Cysteine (mg 100 g^−1^)	13.41 ± 0.69	86.86 ± 4.45
Available carbohydrates (g 100 g^−1^)	3.97 ± 0.02	25.69 ± 0.11
Nitrogen-free extract (g 100 g^−1^)	8.01 ± 0.01	51.89 ± 0.09
Total carbohydrates (includes fiber) (g 100 g^−1^)	9.16 ± 0.01	59.36 ± 0.07
Energy values (kcal 100 g^−1^)	38.87 ± 0.07	251.76 ± 0.48
Energy values (kJ/100 g)	162.72 ± 0.31	1054.08 ± 2.01

**Table 3:** Fatty acids composition of *D. tenuifolia* leaves expressed as relative percentages.

Fatty Acids Composition	*D. tenuifolia* Leaves
Palmitic acid (C16:0)	13.27 ± 0.12
Margaric acid (C17:0)	7.25 ± 0.09
Stearic acid (C18:0)	1.02 ± 0.04
Oleic acid (C18:1)	16.04 ± 0.09
Linoleic acid (C18:2 n-6)	16.48 ± 0.08
α-Linolenic acid (C18:3 n-3)	38.22 ± 0.08
Arachidic acid (C20:0)	6.20 ± 0.09
Total	98.48 ± 0.05
SFA-saturated fatty acids	43.78 ± 0.11
MUFA-monounsaturated fatty acids	16.48 ± 0.08
PUFA-polyunsaturated fatty acids	44.42 ± 0.16

**Table 4:** Total phenolic and flavonoid contents in different *D. tenuifolia* extracts, expressed per gram of extract, are shown as mean ± standard deviation.

Type of Extract	Total Phenolic Compounds	Total Flavonoid Compounds
	mg eq. gallic acid g^−1^	mg eq. quercetin g^−1^
Boiling in methanol (80%)	125.41 ± 0.92	3.72 ± 0.06
Maceration in ethanol (100%)	35.80 ± 0.30	1.21 ± 0.03
Maceration in ethanol (50%)	23.46 ± 0.47	0.72 ± 0.02
Maceration in water (100%)	20.25 ± 0.44	0.64 ± 0.02
Infusion	20.09 ± 0.10	0.94 ± 0.03

**Table 5:** Compounds identified in the extract of *D. tenuifolia* leaves obtained by boiling it in 80% methanol using HPLC-PDA-ESI-MS^n^.

No.	Rt (min)	λmax (nm)	ESI-MS^n^ [*m*/*z* (Relative Abundance. %)]			Attempt to Identify [Reference]
			Precursor Ion [M − H]^−^/[M + H]^+^	MS^2^	MS^3^	
1	1.74	-	[M − H]^−^ 209	209(100); 165(55); 181(20); 191(10)	-	Glucaric acid [33]
2	1.90	-	[M − H]^−^ 510	430(100); 422(90); 372(20)	268(100); 250(40); 234(30)	Sulfoglucobrassicin
			[M + H]^+^ 513	175(100); 450(80); 352(60); 369(50); 337(30)	175(100)	
3	1.94	-	[M + H]^+^ 123	123(100); 105(5)	123(100); 105(5)	Benzoic acid [34]
4	2.04	-	[M − H]^−^ 436	372(100)	259(100); 275(30) 194 (30);	Glucoraphanin [19,35,36]
5	2.12	-	[M + H]^+^ 116	116(100)	116(100)	Proline [37]
6	2.89	-	[M + H]^+^ 130	130(100); 84(10)	130(100); 84(10)	Pipecolic acid [38]
7	2.95	-	[M + H]^+^ 167	167(5); 149(35); 126(100)	126(100); 85(10)	Dihidro-coumaric acid (phloretic acid) [39,40]
8	3.34	-	[M − H]^−^ 494	414(100)	252(100)	6-methylsulfonyl-3-oxohexyl- glucosinolate [19,41]
9	27.50	266; 353	[M − H]^−^ 833 *	787(100); 574(40); 760(35)	-	Quercetin-3-*O*-hexoside- dihexoside [19]
			[M + H]^+^ 789	465(100); 627(95); 303(50)	-	
10	29.43	269, 330	[M − H]^−^ 609	609(45); 447(80); 285(100)	285(100)	Kaempferol-3-*O*-dihexoside [19,42]
			[M − H]^+^ 611	449(100); 287(25)	287(100)	
11	29.94	270, 350	[M + H]^+^ 641	479(100); 317(50)	317(100); 302(10)	Rhamnetin-3-*O*-dihexose [42,43]
12	30.63	271; 353	[M − H]^−^ 993	831(100)	669(100); 463(30); 301(5)	Quercetin-3,4′-diglucoside-3′-(6-sinapoyl-glucoside) [6,19]
			[M + H]^+^ 995	976(100); 832(80); 671(30)	-	
13	30.86	255; 265sh; 353	[M − H]^−^ 609	609(100); 301(100)	301(100); 179(40); 151(30)	Quercetin-3-*O*-deoxyhexose- hexose [19,42]
			[M + H]^+^ 611	465(40); 303(100)	303(100)	
14	34.18	-	[M + H]^+^ 1019 *	1002(100)	958(100); 913(95)	Quercetin-3,4′-di-glucoside-3′-(6-methoxycaffeoyl-glucoside) [8]
15	37.43	-	[M − H]^−^ 347	329(100); 347(50)	311(100); 293(45); 201(35); 171(30); 129(30)	9,10,12,13-tetrahydroxy-octadecanoic acid (Sativic acid) [44]
16	44.46	-	[M − H]^−^ 329	329(100); 311(80)	293(100); 211(50); 171(40); 311(30); 275(20)	9,12,13-trihydroxyoctadec-10-enoic acid [42]
17	44.82	-	[M − H]^−^ 311	311(100); 293(5)	-	Eicosanoic acid (Arachidic acid) [19,45]
18	50.43	255; 380	[M − H]^−^ 315	297(100) 315(50)	279(100) 171(90)	Isorhamnetin [46]
19	62.15	-	[M − H]^−^ 1199	1137(100)	-	Quercetin-3-(2-sinapoyl-glucoside)-3′-(6-sinapoyl-glucoside)-4′-glucoside [8,10]
20	63.94	-	[M − H]^−^ 1169	1007(100)	-	Quercetin-3-(2-feruloyl-glucoside)-3′-(6-sinapoyl-glucoside)-4′-glucoside [8,10]

*: adduct; sh: shoulder; λmax.: maximum wavelength in UV–Vis spectrum.

**Table 6:** Antioxidant activity of various extracts from *D. tenuifolia* (mean ± standard deviation).

Type of Extract	ABTS			DPPH		FRAP	
	mg eq. ascorbic acid g^−1^	mg eq. trolox g^−1^	IC_50_ (µg mL^−1^)	mg eq. trolox g^−1^	IC_50_ (µg mL^−1^)	µmol eq. Fe(II) g^−1^	µmol eq. trolox g^−1^
Boiling in methanol (80%)	47.68 ± 0.51	39.43 ± 0.97	57.54 ± 0.18	12.96 ± 0.02	302.73 ± 2.36	752.71 ± 4.59	257.15 ± 1.65
Maceration in ethanol (100%)	10.41 ± 0.45	8.61 ± 0.86	159.44 ± 5.67	3.49 ± 0.11	771.44 ± 8.10	391.62 ± 1.29	138.88 ± 0.54
Maceration in ethanol (50%)	11.47 ± 0.28	12.23 ± 0.53	151.44 ± 3.09	4.83 ± 0.05	647.77 ± 5.96	289.27 ± 1.52	100.80 ± 0.63
Maceration in water (100%)	11.80 ± 0.22	10.20 ± 0.42	141.56 ± 2.11	3.24 ± 0.05	813.35 ± 9.40	210.80 ± 1.69	66.30 ± 0.70
Infusion	12.73 ± 0.33	14.25 ± 0.60	137.93 ± 3.90	3.99 ± 0.05	698.83 ± 6.20	270.52 ± 1.72	86.14 ± 0.72
Control—Ascorbic acid	-	-	2.81 ± 0.02	-	-	-	-
Control—Trolox	-	-	2.25 ± 0.01	-	3.02 ± 0.02	-	-

## Data Availability

Data is contained within the article.

## Appendix A

Table A1 and Table A2 in Appendix A present the results of the soil and water analyses conducted prior to the establishment of the *D. tenuifolia* crop.

**Table A1:** Soil analyses.

Parameters	Soil Composition
Field texture	Medium
Fine soil (Φ < 2 mm) (%)	85.21 ± 0.32
Organic matter (%)	3.80 ± 0.10
pH (H_2_O)	7.96 ± 0.02
Electrical conductivity (µS cm^−1^)	298.47 ± 3.72
Available phosphorus (mg P_2_O_5_ kg^−1^)	2032.13 ± 41.84
Available potassium (mg K_2_O kg^−1^)	209.80 ± 2.47
Available ion (mg Fe kg^−1^)	119.20 ± 2.41
Available copper (mg Cu kg^−1^)	50.60 ± 2.01
Available zinc (mg Zn kg^−1^)	47.50 ± 1.87
Available manganese (mg Mn kg^−1^)	32.70 ± 0.54
Available boron (mg B kg^−1^)	0.46 ± 0.02
Exchangeable cations—Potassium (meq. K^+^ 100 g^−1^)	0.42 ± 0.09
Exchangeable cations—Sodium (meq. Na^+^ 100 g^−1^)	0.10 ± 0.02
Exchangeable cations—Calcium (meq. Ca^2+^ 100 g^−1^)	11.01 ± 0.41
Exchangeable cations—Magnesium (meq. Mg^2+^ 100 g^−1^)	1.27 ± 0.21
Total Calcium (%)	4.02 ± 0.08
Total Potassium (%)	0.55 ± 0.04
Total Nitrogen (%)	0.21 ± 0.02
Total Phosphorus (%)	0.19 ± 0.02
Total Magnesium (%)	0.14 ± 0.02
Total Sodium (%)	0.12 ± 0.02
Total Zinc (mg Zn kg^−1^)	150.93 ± 1.14
Total Copper (mg Cu kg^−1^)	134.54 ± 1.21
Total Boron (mg B kg^−1^)	45.52 ± 1.26
Total Lead (mg Pb kg^−1^)	20.01 ± 0.42
Total Chromium (mg Cr kg^−1^)	9.45 ± 0.18
Total Nickel (mg Ni kg^−1^)	7.13 ± 0.09
Total Cadmium (mg Cd kg^−1^)	<1.03 (L.O.Q)
Total Mercury (mg Hg kg^−1^)	0.04 ± 0.00
L.O.Q: Limit of Quantification	

**Table A2:** Irrigation water analyses.

Parameters	Water Composition
pH (20 °C)	7.3 ± 0.0
Electrical conductivity (µS cm^−1^. 20 °C)	495.8 ± 0.8
Total alkalinity mg L^−1^ (mg CaCO_3_ L^−1^)	26.9 ± 0.2
Oxidability (mg O_2_ L^−1^)	0.2 ± 0.0
Total hardness (mg CaCO_3_ L^−1^)	2.6 ± 0.1
Chlorides (mg L^−1^)	59.3 ± 0.3
Nitrates (mg L^−1^)	1.98 ± 0.1
Nitrites (mg L^−1^)	0.1 ± 0.0
Sulfates (mg L^−1^)	44.2 ± 0.2
Ammoniacal Nitrogen (mg L^−1^)	0.1 ± 0.0
Calcium (mg L^−1^)	18.9 ± 0.2
Iron (mg L^−1^)	0.2 ± 0.0
Magnesium (mg L^−1^)	15.4 ± 0.1
Potassium (mg L^−1^)	14.1 ± 0.1
Sodium (mg L^−1^)	8.1 ± 0.2

## References

[1] Warwick S.I., Schmidt R., Bancroft I. (2011). Brassicaceae in Agriculture. Genetics and Genomics of the Brassicaceae.

[2] WFO Plant List|World Flora Online. https://wfoplantlist.org/taxon/wfo-7000000082-2024-06?utm_source=chatgpt.com.

[3] Caruso G., Parrella G., Giorgini M., Nicoletti R. (2018). Crop Systems, Quality and Protection of *Diplotaxis tenuifolia*. Agriculture.

[4] Ressurreição S., Salgueiro L., Figueirinha A. (2024). *Diplotaxis* Genus: A Promising Source of Compounds with Nutritional and Biological Properties. Molecules.

[5] Bennett R.N., Rosa E.A.S., Mellon F.A., Kroon P.A. (2006). Ontogenic Profiling of Glucosinolates, Flavonoids, and Other Secondary Metabolites in *Eruca sativa* (Salad Rocket), Diplotaxis Erucoides (Wall Rocket), *Diplotaxis tenuifolia* (Wild Rocket), and Bunias Orientalis (Turkish Rocket). J. Agric. Food Chem..

[6] Bell L., Oruna-Concha M.J., Wagstaff C. (2015). Identification and Quantification of Glucosinolate and Flavonol Compounds in Rocket Salad (Eruca Sativa, *Eruca vesicaria* and *Diplotaxis tenuifolia*) by LC–MS: Highlighting the Potential for Improving Nutritional Value of Rocket Crops. Food Chem..

[7] Martínez-Sánchez A., Llorach R., Gil M.I., Ferreres F. (2007). Identification of New Flavonoid Glycosides and Flavonoid Profiles To Characterize Rocket Leafy Salads (*Eruca vesicaria* and *Diplotaxis tenuifolia*). J. Agric. Food Chem..

[8] Pasini F., Verardo V., Caboni M.F., D’Antuono L.F. (2012). Determination of Glucosinolates and Phenolic Compounds in Rocket Salad by HPLC-DAD–MS: Evaluation of *Eruca sativa* Mill. and *Diplotaxis tenuifolia* L. Genetic Resources. Food Chem..

[9] Bell L., Wagstaff C. (2014). Glucosinolates, Myrosinase Hydrolysis Products, and Flavonols Found in Rocket (*Eruca sativa* and *Diplotaxis tenuifolia*). J. Agric. Food Chem..

[10] Kilibarda S., Milinčić D.D., Vuković S., Pešić M.B., Jelačić S., Moravčević Đ., Kostić A.Ž. (2025). Untargeted HPLC Metabolomic Profiling and Antioxidant Activity of Wild Rocket’s (*Diplotaxis tenuifolia* (L.) DC.) Edible Leaves Influenced by Biofortification, Hybrid Varieties and Harvest Time. Food Biosci..

[11] Hall M.K.D., Jobling J.J., Rogers G.S. (2015). Variations in the Most Abundant Types of Glucosinolates Found in the Leaves of Baby Leaf Rocket under Typical Commercial Conditions. J. Sci. Food Agric..

[12] Raffo A., Baiamonte I., De Nicola G.R., Melini V., Moneta E., Nardo N., Peparaio M., Saggia Civitelli E., Sinesio F. (2024). Sensory Attributes Driving Preference for Wild Rocket (*Diplotaxis tenuifolia*) Leaves Tasted as a Single Ingredient and as a Part of a Recipe. Foods.

[13] Conforti F., Perri V., Menichini F., Marrelli M., Uzunov D., Statti G.A., Menichini F. (2012). Wild Mediterranean Dietary Plants as Inhibitors of Pancreatic Lipase. Phytother. Res..

[14] Durazzo A., Azzini E., Lazzè M.C., Raguzzini A., Pizzala R., Maiani G. (2013). Italian Wild Rocket *Diplotaxis tenuifolia* (L.) DC.: Influence of Agricultural Practices on Antioxidant Molecules and on Cytotoxicity and Antiproliferative Effects. Agriculture.

[15] Jeong H.-R., Kim S., Oh R., Lee Y., Choi J., Kim M.C. (2025). Comparative Evaluation of the Antioxidant Activity, and Anti-Melanogenic Properties of *Diplotaxis tenuifolia* and *Eruca sativa* Extracts on B16F10 Melanoma Cells. J. Korean Soc. Food Sci. Nutr..

[16] Romano R., Pizzolongo F., Luca L.D., Cozzolino E., Rippa M., Ottaiano L., Mormile P., Mori M., Mola I.D. (2022). Bioactive Compounds and Antioxidant Properties of Wild Rocket (*Diplotaxis tenuifolia* L.) Grown under Different Plastic Films and with Different UV-B Radiation Postharvest Treatments. Foods.

[17] Gisbert C., Clemente R., Navarro-Aviñó J., Baixauli C., Ginér A., Serrano R., Walker D.J., Bernal M.P. (2006). Tolerance and Accumulation of Heavy Metals by Brassicaceae Species Grown in Contaminated Soils from Mediterranean Regions of Spain. Environ. Exp. Bot..

[18] Official Journal of the European Union (2023). Commission Regulation (EU) 2023/915 of 25 April 2023 on Maximum Levels for Certain Contaminants in Food and Repealing Regulation (EC) No 1881/2006 (Text with EEA Relevance).

[19] Ressurreição S., Salgueiro L., Figueirinha A. (2025). Diplotaxis Muralis as an Emerging Food Crop: Chemical Composition, Nutritional Profile and Antioxidant Activities. Plants.

[20] Official Journal of the European Union (2011). Brussels, Belgium Regulation (EU) No 1169/2011 of the European Parliament and of the Council of 25 October 2011 on the Provision of Food Information to Consumers (Text with EEA Relevance).

[21] Instituto Nacional de Saúde Doutor Ricardo Jorge (INSA) Plataforma Portuguesa de Informação Alimentar (PortFIR). https://portfir.insa.min-saude.pt/pt/.

[22] Fukalova T., García Martínez M.D., Raigón M.D. (2021). Five Undervalued Edible Species Inherent to Autumn-Winter Season: Nutritional Composition, Bioactive Constituents and Volatiles Profile. PeerJ.

[23] Jdir H., Khemakham B., Chakroun M., Zouari S., Ali Y.B., Zouari N. (2015). *Diplotaxis simplex* Suppresses Postprandial Hyperglycemia in Mice by Inhibiting Key-Enzymes Linked to Type 2 Diabetes. Rev. Bras. Farmacogn..

[24] Pimpini F., Giannini M., Lazzarin R. (2005). Ortaggi Da Foglia e Da Taglio.

[25] Koutsoukis C., Roukos C., Demertzis P.G., Kandrelis S., Akrida-Demertzi K. (2019). The Variation of the Chemical Composition of the Main Plant Species in a Subalpine Grassland in Northwestern Greece. Legume Sci..

[26] Guan Z.-W., Yu E.-Z., Feng Q. (2021). Soluble Dietary Fiber, One of the Most Important Nutrients for the Gut Microbiota. Molecules.

[27] Nurzynska-Wierdak R. (2015). Protein Nutritional Value of Rocket Leaves and Possibilities of Itsmodification during Plant Growth. Turk. J. Agric. For..

[28] Lisiewska Z., Kmiecik W., Korus A. (2008). The Amino Acid Composition of Kale (*Brassica oleracea* L. var. *Acephala*), Fresh and after Culinary and Technological Processing. Food Chem..

[29] Murcia M.A., López-Ayerra B., Martínez-Tomé M., García-Carmona F. (2001). Effect of Industrial Processing on Amino Acid Content of Broccoli. J. Sci. Food Agric..

[30] Salah N.B., Casabianca H., Jannet H.B., Chenavas S., Sanglar C., Fildier A., Bouzouita N. (2015). Phytochemical and Biological Investigation of Two Diplotaxis Species Growing in Tunisia: *D. virgata* & *D. erucoides*. Molecules.

[31] Lunn J., Theobald H.E. (2006). The Health Effects of Dietary Unsaturated Fatty Acids. Nutr. Bull..

[32] Falleh H., Msilini N., Oueslati S., Ksouri R., Magne C., Lachaâl M., Karray-Bouraoui N. (2013). *Diplotaxis harra* and *Diplotaxis simplex* Organs: Assessment of Phenolics and Biological Activities before and after Fractionation. Ind. Crops Prod..

[33] Arora R. (2024). Glucosinolates and Their Hydrolytic Products—A Love Story of Environmental, Biological, and Chemical Conditions. J. AOAC Int..

[34] Xu S., Pavlov J., Attygalle A.B. (2017). Collision-Induced Dissociation Processes of Protonated Benzoic Acid and Related Compounds: Competitive Generation of Protonated Carbon Dioxide or Protonated Benzene. J. Mass Spectrom..

[35] Fabre N., Poinsot V., Debrauwer L., Vigor C., Tulliez J., Fourasté I., Moulis C. (2007). Characterisation of Glucosinolates Using Electrospray Ion Trap and Electrospray Quadrupole Time-of-Flight Mass Spectrometry. Phytochem. Anal..

[36] Dong M., Tian Z., Ma Y., Yang Z., Ma Z., Wang X., Li Y., Jiang H. (2021). Rapid Screening and Characterization of Glucosinolates in 25 Brassicaceae Tissues by UHPLC-Q-Exactive Orbitrap-MS. Food Chem..

[37] Choi S.-S., Song M.J., Kim O.-B., Kim Y. (2013). Fragmentation Patterns of Protonated Amino Acids Formed by Atmospheric Pressure Chemical Ionization. Rapid Commun. Mass Spectrom..

[38] El Sayed Z.I., Hassan W.H.B., Abdel-Aal M.M., Al-Massarani S.M., Abdel-Mageed W.M., Basudan O.A., Parveen M., Abdelsalam E., Abdelaziz S. (2024). Chemical and Biological Characterization of the Ethyl Acetate Fraction from the Red Sea Marine Sponge *Hymedesmia* sp.. Pharmaceuticals.

[39] Schwarz M., Weber F., Durán-Guerrero E., Castro R., del Carmen Rodríguez-Dodero M., García-Moreno M.V., Winterhalter P., Guillén-Sánchez D. (2021). HPLC-DAD-MS and Antioxidant Profile of Fractions from Amontillado Sherry Wine Obtained Using High-Speed Counter-Current Chromatography. Foods.

[40] Tong Z., Li W., Jiang J., Wang C. (2025). Effects of Steam Explosion-Assisted Extraction on the Structural Characteristics, Phenolic Profile, and Biological Activity of Valonea. Foods.

[41] Cataldi T.R.I., Lelario F., Orlando D., Bufo S.A. (2010). Collision-Induced Dissociation of the A + 2 Isotope Ion Facilitates Glucosinolates Structure Elucidation by Electrospray Ionization-Tandem Mass Spectrometry with a Linear Quadrupole Ion Trap. Anal. Chem..

[42] Loizzo M.R., Napolitano A., Bruno M., Geraci A., Schicchi R., Leporini M., Tundis R., Piacente S. (2021). LC-ESI/HRMS Analysis of Glucosinolates, Oxylipins and Phenols in Italian Rocket Salad (*Diplotaxis erucoides* subsp. *Erucoides* (L.) DC.) and Evaluation of Its Healthy Potential. J. Sci. Food Agric..

[43] Jiang C., Gates P.J. (2024). Systematic Characterisation of the Fragmentation of Flavonoids Using High-Resolution Accurate Mass Electrospray Tandem Mass Spectrometry. Molecules.

[44] Ribeiro da Silva Lima L., Barros Santos M.C., Gomes P.W.P., Fernández-Ochoa Á., Simões Larraz Ferreira M. (2024). Overview of the Metabolite Composition and Antioxidant Capacity of Seven Major and Minor Cereal Crops and Their Milling Fractions. J. Agric. Food Chem..

[45] Islam A.K.M.M., Hong S.-M., Lee H.-S., Moon B.-C., Kim D., Kwon H. (2018). Identification and Characterization of Matrix Components in Spinach during QuEChERS Sample Preparation for Pesticide Residue Analysis by LC–ESI–MS/MS, GC–MS and UPLC-DAD. J. Food Sci. Technol..

[46] Jdir H., Kolsi R.B.A., Zouari S., Hamden K., Zouari N., Fakhfakh N. (2017). The Cruciferous Diplotaxis Simplex: Phytochemistry Analysis and Its Protective Effect on Liver and Kidney Toxicities, and Lipid Profile Disorders in Alloxan-Induced Diabetic Rats. Lipids Health Dis..

[47] Tripodi P., Francese G., Mennella G. (2017). Rocket Salad: Crop Description, Bioactive Compounds and Breeding Perspectives. Adv. Hortic. Sci..

[48] Oliviero T., Verkerk R., Dekker M. (2018). Isothiocyanates from Brassica Vegetables—Effects of Processing, Cooking, Mastication, and Digestion. Mol. Nutr. Food Res..

[49] Melrose J. (2019). The Glucosinolates: A Sulphur Glucoside Family of Mustard Anti-Tumour and Antimicrobial Phytochemicals of Potential Therapeutic Application. Biomedicines.

[50] Williams D.E. (2021). Indoles Derived From Glucobrassicin: Cancer Chemoprevention by Indole-3-Carbinol and 3,3′-Diindolylmethane. Front. Nutr..

[51] Savchenko T., Walley J.W., Chehab E.W., Xiao Y., Kaspi R., Pye M.F., Mohamed M.E., Lazarus C.M., Bostock R.M., Dehesh K. (2010). Arachidonic Acid: An Evolutionarily Conserved Signaling Molecule Modulates Plant Stress Signaling Networks. Plant Cell.

[52] Berg-Falloure K.M., Kolomiets M.V., Berg-Falloure K.M., Kolomiets M.V. (2023). Ketols Emerge as Potent Oxylipin Signals Regulating Diverse Physiological Processes in Plants. Plants.

[53] Savchenko T., Degtyaryov E., Radzyukevich Y., Buryak V., Savchenko T., Degtyaryov E., Radzyukevich Y., Buryak V. (2022). Therapeutic Potential of Plant Oxylipins. Int. J. Mol. Sci..

[54] Ahmed A.F., Wen Z.-H., Bakheit A.H., Basudan O.A., Ghabbour H.A., Al-Ahmari A., Feng C.-W. (2022). A Major Diplotaxis Harra-Derived Bioflavonoid Glycoside as a Protective Agent against Chemically Induced Neurotoxicity and Parkinson’s Models; In Silico Target Prediction; and Biphasic HPTLC-Based Quantification. Plants.

[55] Bahloul N., Bellili S., Aazza S., Chérif A., Faleiro M.L., Antunes M.D., Miguel M.G., Mnif W. (2016). Aqueous Extracts from Tunisian Diplotaxis: Phenol Content, Antioxidant and Anti-Acetylcholinesterase Activities, and Impact of Exposure to Simulated Gastrointestinal Fluids. Antioxidants.

[56] Ferreira C.S., Veiga A., Caetano A., Gonzalez-Pelayo O., Karine-Boulet A., Abrantes N., Keizer J., Ferreira A.J. (2020). Assessment of the Impact of Distinct Vineyard Management Practices on Soil Physico-Chemical Properties. Air Soil Water Res..

[57] Clescari L.S., Greenberg A.E., Eaton A.D. (1998). Standard Methods for the Examination of Water and Wastewater.

[58] Cunniff P. (1997). Official Methods of Analysis of AOAC International.

[59] Horwitz W. (2002). Official Methods of Analysis of AOAC International.

[60] Van Soest P.J., Robertson J.B., Lewis B.A. (1991). Methods for Dietary Fiber, Neutral Detergent Fiber, and Nonstarch Polysaccharides in Relation to Animal Nutrition. J. Dairy Sci..

[61] Food and Agriculture Organization of the United Nations (2003). Food Energy—Methods of Analysis and Conversion Factors.

[62] Tacon A.G.J. (1987). The Nutrition and Feeding of Farmed Fish and Shrimp—A Training Manual—2. Nutrient Sources and Composition.

[63] Mota C., Santos M., Mauro R., Samman N., Matos A.S., Torres D., Castanheira I. (2016). Protein Content and Amino Acids Profile of Pseudocereals. Food Chem..

[64] Assunção M.F.G., Varejão J.M.T.B., Santos L.M.A. (2017). Nutritional Characterization of the Microalga *Ruttnera lamellosa* Compared to *Porphyridium purpureum*. Algal Res..

[65] Gião M.S., González-Sanjosé M.L., Rivero-Pérez M.D., Pereira C.I., Pintado M.E., Malcata F.X. (2007). Infusions of Portuguese Medicinal Plants: Dependence of Final Antioxidant Capacity and Phenol Content on Extraction Features. J. Sci. Food Agric..

[66] Al-Dabbas M.M., Suganuma T., Kitahara K., Hou D.-X., Fujii M. (2006). Cytotoxic, Antioxidant and Antibacterial Activities of *Varthemia iphionoides* Boiss. Extracts. J. Ethnopharmacol..

[67] Brand-Williams W., Cuvelier M.E., Berset C. (1995). Use of a Free Radical Method to Evaluate Antioxidant Activity. LWT-Food Sci. Technol..

[68] Pulido R., Bravo L., Saura-Calixto F. (2000). Antioxidant Activity of Dietary Polyphenols As Determined by a Modified Ferric Reducing/Antioxidant Power Assay. J. Agric. Food Chem..

[69] Pedreiro S., da Ressurreição S., Lopes M., Cruz M.T., Batista T., Figueirinha A., Ramos F. (2021). *Crepis vesicaria* L. Subsp. *taraxacifolia* Leaves: Nutritional Profile, Phenolic Composition and Biological Properties. Int. J. Environ. Res. Public Health.

